# Linking *in silico* MS/MS spectra with chemistry data to improve identification of unknowns

**DOI:** 10.1038/s41597-019-0145-z

**Published:** 2019-08-02

**Authors:** Andrew D. McEachran, Ilya Balabin, Tommy Cathey, Thomas R. Transue, Hussein Al-Ghoul, Chris Grulke, Jon R. Sobus, Antony J. Williams

**Affiliations:** 1 Oak Ridge Institute for Science and Education (ORISE) Research Participation Program, United States Environmental Protection Agency, 109 T.W. Alexander Dr., Research Triangle Park, Durham, NC 27711 USA; 2 National Center for Computational Toxicology, Office of Research and Development, U.S. Environmental Protection Agency, 109 T.W. Alexander Dr., Research Triangle Park, Durham, NC 27711 USA; 3 CSRA Inc., 109 T.W. Alexander Drive, Research Triangle Park, Durham, NC 27711 USA; 4 GDIT, 109 T.W. Alexander Dr., Research Triangle Park, Durham, NC 27711 USA; 5 Oak Ridge Associated Universities (ORAU), 109 T.W. Alexander Dr., Research Triangle Park, Durham, NC 27711 USA; 6 National Exposure Research Laboratory, Office of Research and Development, U.S. Environmental Protection Agency, 109 T.W. Alexander Dr., Research Triangle Park, Durham, NC 27711 USA

**Keywords:** Cheminformatics, Databases, Mass spectrometry

## Abstract

Confident identification of unknown chemicals in high resolution mass spectrometry (HRMS) screening studies requires cohesive workflows and complementary data, tools, and software. Chemistry databases, screening libraries, and chemical metadata have become fixtures in identification workflows. To increase confidence in compound identifications, the use of structural fragmentation data collected via tandem mass spectrometry (MS/MS or MS^2^) is vital. However, the availability of empirically collected MS/MS data for identification of unknowns is limited. Researchers have therefore turned to *in silic*o generation of MS/MS data for use in HRMS-based screening studies. This paper describes the generation *en masse* of predicted MS/MS spectra for the entirety of the US EPA’s DSSTox database using competitive fragmentation modelling and a freely available open source tool, CFM-ID. The generated dataset comprises predicted MS/MS spectra for ~700,000 structures, and mappings between predicted spectra, structures, associated substances, and chemical metadata. Together, these resources facilitate improved compound identifications in HRMS screening studies. These data are accessible via an SQL database, a comma-separated export file (.csv), and EPA’s CompTox Chemicals Dashboard.

## Abstract (summary)

**Table:** 

Design Type(s)	chemical structure classification objective • modeling and simulation objective • mass spectrometry data analysis objective
Measurement Type(s)	tandem mass spectrometry
Technology Type(s)	digital curation
Factor Type(s)	chemical entity
Sample Characteristic(s)

Machine-accessible metadata file describing the reported data (ISA-Tab format)

## Background & Summary

The rapid identification of small molecules in metabolomics, exposomics, and environmental monitoring studies increasingly involves the use of high resolution mass spectrometry (HRMS) and non-targeted analysis (NTA) techniques^1–3^. NTA experiments generally incorporate complementary software (open and commercial tools) and chemistry databases to enable effective and accurate compound identification^4–6^. Different instrumentation and NTA approaches require different tools for effective annotation. For example, compound identification strategies based on MS^1^ data (yielding exact mass and molecular formula) typically rely on chemical metadata (e.g. the number of data sources or literature references linked to a chemical)^6^, whereas those based on MS/MS data (yielding MS^1^ data and fragment ions) are enhanced by matching of empirical fragmentation spectra with library spectra^7,8^. Recent studies have shown that melding of these approaches leads to improved identification accuracy over spectral matching alone^7,9^. Thus, coupling robust metadata with spectral matching capabilities must now be the focus of research efforts to maximize yield from NTA identification techniques.

When considering the number of known compounds in public databases, the availability of library MS/MS spectra is quite limited^2,10^. Open spectral libraries such as MassBank (https://massbank.eu/MassBank/)^11^, MoNA (http://mona.fiehnlab.ucdavis.edu/), METLIN^12^, and mzCloud (https://www.mzcloud.org/) are rich with empirical spectra, but do not yet fully cover the broad chemical space that may be monitored in NTA studies. Instrument vendors further provide empirical spectral data for users to purchase (with matching algorithms executed within vendor software), but access and coverage remains limited^13^. To address these gaps, researchers have developed *in silico* fragmenters and MS/MS prediction models, including MetFrag^7^, CSI Finger-ID^14^, and CFM-ID^8^, among a number of others available commercially (e.g. ACD/MS Fragmenter^15^, Mass Frontier^16^). Use of predicted MS/MS spectra in identification workflows has proven effective^5^, but requires the incorporation of command line utilities and/or on-the-fly processing of data for single chemicals. Prediction of MS/MS spectra *en masse* and mapping pre-computed spectra to structures and metadata within chemistry databases can enhance identification schemes and enable integration into various software systems and workflows.

**Fig. 1:**
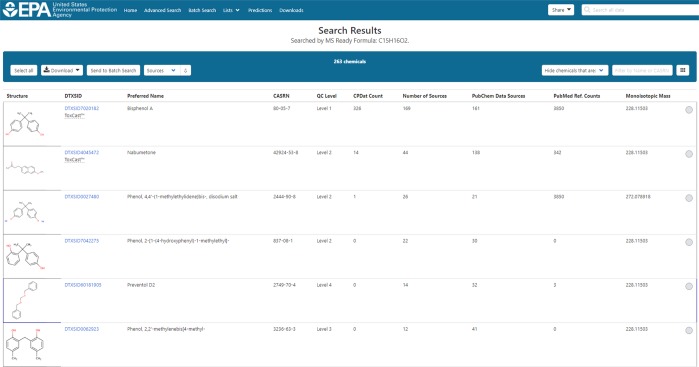
Search results from an MS-Ready Formula search of C_15_H_16_O_2_ Candidate chemical structures are denoted by a DTXSID and preferred name and contain linked metadata such as the Number of Sources, CPDat Count, and PubMed Ref. Count. Rank ordering by metadata brings the most likely chemicals to the top of the search results list.

The US EPA’s DSSTox database is a comprehensive chemistry resource, containing more than 760,000 distinct chemical substances, associated chemical structures, and metadata^17^, and serves as the underpinning for EPA’s CompTox Chemicals Dashboard (https://comptox.epa.gov/dashboard)^18^. Among its many functionalities, the Dashboard enables searching of masses and formulae generated from HRMS experiments. The data and algorithms associated with Dashboard searching have been shown to outperform the much larger ChemSpider database (ca. 67 million chemicals as of July 2018) using data source ranking for the identification of unknowns^6^. As an example, consider a search for the formula C_15_H_16_O_2_ which produces a total of 263 results. Rank ordering the results based on data source or literature reference counts brings the most likely chemical (Bisphenol A) to the top of the search results (Fig. 1).

Additional metadata are now being optimized in a combined ranking scheme to further improve identifications. To improve Dashboard capabilities that support NTA research, we are generating, storing, and mapping predicted MS/MS spectra for all structures in the database.

Herein we describe: (1) the generation and storage of predicted MS/MS spectra for all chemical structures contained with DSSTox; (2) the validation and mapping of spectra to structures and substances; and (3) the publication of the comprehensive dataset for public dissemination (including the complete SQL database and schema). MS/MS spectra were predicted using competitive fragmentation modelling (CFM) and the open command line tools developed by Allen *et al*.^8,19,20^ and named CFM-ID (available here: http://sourceforge.net/projects/cfm-id). All remaining data are sourced from the US EPA’s DSSTox database and available via the EPA’s CompTox Chemicals Dashboard (https://comptox.epa.gov/dashboard). Open and accessible data, integrated and provided in this dataset, enables NTA practitioners an improved means of small molecule identification when using MS/MS data from HRMS experiments.

## Methods

### Generation of predicted MS/MS Data

To maximize use of predicted MS/MS data, both for our processes^13,21^ and the mass spectral community at large, “MS-Ready” structures were used in the prediction model. An MS-Ready structure represents the form of a structure that would be observed via HRMS; these structures are de-salted, de-solvated, and processed such that chemical mixtures are separated^22^. These structures are stored in the DSSTox database with unique chemical identifiers (DTXCIDs) and linked to unique substance identifiers (DTXSIDs) to enable use of the structures and associated substance-level metadata in HRMS applications.

MS/MS spectra were predicted using CFM-ID with pre-trained parameters as defined by CFM-ID literature and described by Allen *et al*.^8,19,20^. All source code was downloaded from the CFM-ID SourceForge site: http://sourceforge.net/projects/cfm-id. The input data were 843,113 MS-Ready chemical structures as SMILES strings. Additional data associated with chemical structures included DTXCIDs, molecular formulas, standard InChIKeys generated using the Indigo Toolkit (http://lifescience.opensource.epam.com/indigo/), and monoisotopic masses. The obtained chemicals were saved in a local tab separated file.

MS/MS spectra were generated for each structure in the following ionization modes: electrospray ionization in both positive and negative modes (ESI+ and ESI-, respectively) at three collision energies (Energy0–10 eV, Energy1–20 eV, and Energy2–40 eV), and electron impact ionization (EI). Spectra were predicted using standard parameters provided with the software and available via the CFM-ID SourceForge site with no limits placed on the number of MS/MS spectra calculated for a given structure.

The mass spectra calculations were performed on a large-scale Linux cluster at the US EPA National Computer Center (https://www.epa.gov/greeningepa/national-computer-center). A master shell script was used to generate over 4,000 Slurm (https://slurm.schedmd.com/) queueing system run scripts that calculated EI, ESI+, and ESI- MS/MS spectra for 200 chemicals each. A small fraction of chemicals (<700) was excluded from CFM-ID calculations due to missing data and/or structural issues expected to fail in processing (such as SMILES notations of radicals, e.g. CC(C=C)=C[Al] |^3:5|). An additional 56 chemicals failed during calculation of all three prediction types. This was believed to occur due to the structural constraints of the models and ionization types as many of the failed chemicals were permanently charged species and metals (“Chemical Structures that failed during mass spectral prediction”, data available at 10.23645/epacomptox.7776212.v1)^23^. Mode-specific failures occurred as follows: ~1000 chemicals failed during calculation of EI spectra, ~2000 failed during calculation of ESI+ spectra, and ~18,000 failed during calculation of ESI- spectra. The substantially higher number of failures occurring in ESI- mode are primarily driven by permanently charged species unlikely to ionize in negative electrospray.

For each type of mass spectra (EI, ESI+ and ESI-), the log files were merged and a Python script was used to separate the contents into a final output file (metadata followed by mass spectrum data for each chemical) and an error file (CFM-ID error messages for failed and timed out calculations). The final output file was a .dat ASCII file for each ionization mode (“Predicted EI-MS Spectra of CompTox Chemicals Dashboard Structures”, “Predicted MS/MS Spectra in ESI-positive mode of CompTox Chemicals Dashboard Structures”, “Predicted MS/MS Spectra in ESI-negative mode of CompTox Chemicals Dashboard Structures”, data available at 10.23645/epacomptox.7776212.v1)^23^.

### Data storage and database structure

The raw output of the predicted MS/MS data described above required parsing and manipulation in order to generate MySQL loadable data. A Java application was developed to parse the data and generate MySQL load statements to load the database (described below). The resulting database required ~137 GB of storage and took 10 hours to load.

### Mapping to chemical metadata with DSSTox and associated databases

MS-Ready structures, denoted by individual DTXCIDs, are stored in a structure relationship mapping table linking MS-Ready structures to original DSSTox structures and associated chemical substances (DTXSIDs). Chemical substances are associated with a variety of identifiers (e.g. InChI strings and keys, synonyms, database identifiers) and data (e.g. physicochemical properties, toxicity data, bioactivity data). Additional details regarding the relationship between DTXCIDs and DTXSIDs are explained in more detail elsewhere^18^.

The CompTox Chemicals Dashboard (https://comptox.epa.gov/dashboard/) enables users to search and peruse the data contained within multiple databases (see Table 2 in Williams, *et* *al*.^18^ for a list of all databases). Many of the data contained within these databases are of value for ranking candidate chemicals in search results, including the number of data sources associated with a chemical in PubChem (https://pubchem.ncbi.nlm.nih.gov/), the number of associated articles in PubMed (https://www.ncbi.nlm.nih.gov/pubmed/), and the number of unique consumer product categories associated with a chemical in the Chemical and Products Database (CPDat; https://www.epa.gov/chemical-research/chemical-and-products-database-cpdat)^24^. As discussed above, ranking based on such metadata sources has already proven to be a valuable approach^6^.

To facilitate search and identification of unknowns using HRMS data, an export file from DSSTox was generated to include all DTXCIDs used to generated MS/MS data and valuable metadata, described below. Access to both substance-level metadata and predicted MS/MS data is made possible through the linked DTXCID identifier and database structure.

## Data Records

The data described in this work is available in three primary formats: a SQL relational database, .dat ASCII files containing all predicted spectra, and as a complete export file in comma-separated format (.csv). Two types of data are presented to facilitate compound identification: predicted MS/MS spectral data and chemical metadata, described below and defined as data linked to a chemical structure. Access and use of the data are enabled by the inclusion of unique chemical identifiers (DTXCIDs) within all records to connect chemical structures to their associated data.

### Spectral data

**Fig. 2:**
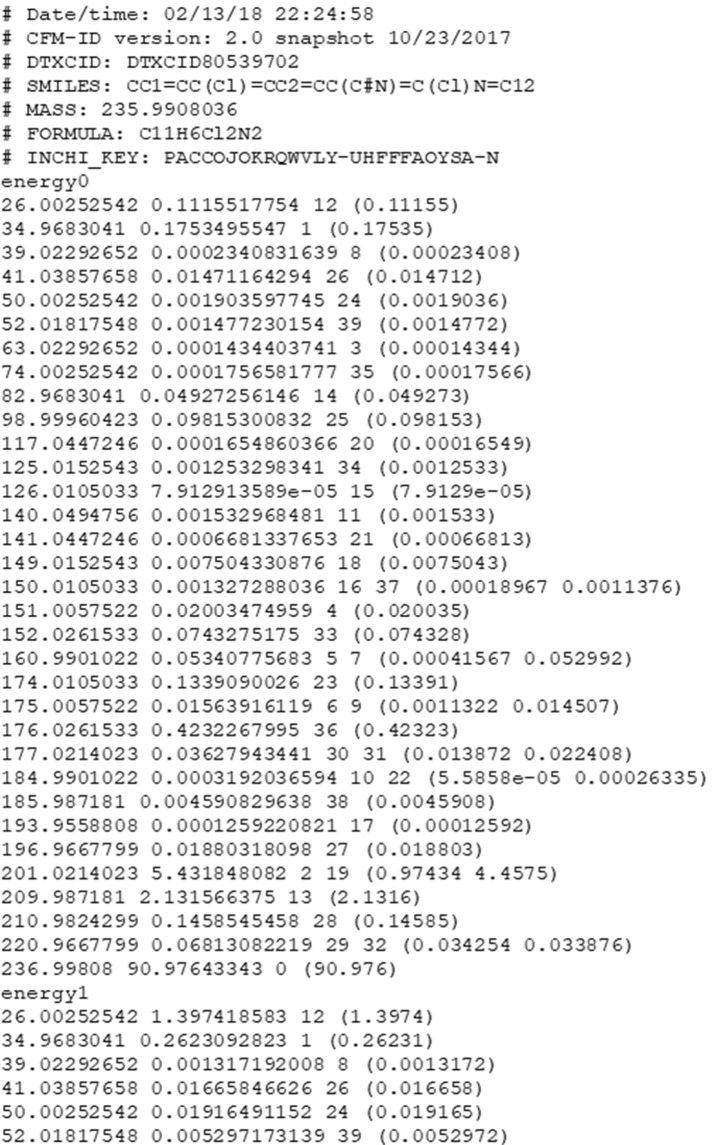
Chemical structure metadata information followed by predicted MS/MS data included in the .dat ASCII prediction files using the example of DTXCID80539702 in ESI-positive mode. Only the first ~50 lines of predictions are shown and structural annotations with SMILES succeeding predictions are not included in the image.

MS/MS spectra were generated for each structure in the following ionization modes: ESI+, ESI-, and EI. Each data record generated for a structure in ESI+ and ESI- contains MS/MS predictions for three collision energy levels while each record for EI contains results from a single collision energy only. Collision energy levels predicted for ESI are as follows: Energy0 (10 eV), Energy1 (20 eV), and Energy2 (40 eV). Preceding spectral predictions for a given structure are the following chemical structure metadata fields (see an example in Fig. 2):

- Date/time: indicating the date and time the prediction was computed
- CFM-ID version: indicating the version of the command line tools
- DTXCID: the unique DSSTox chemical identifier for the structure
- SMILES: the MS-Ready SMILES for the structure
- MASS: the neutral MS-Ready monoisotopic mass
- FORMULA: the MS-Ready molecular formula
- INCHI_KEY: the standard InChI Key for the structure

Immediately following the chemical structure metadata fields are predicted MS/MS fragments in order of collision energy level (Energy0, Energy1, Energy2), when appropriate. Provided within each collision energy level are all fragments generated according to the CFM model, ordered from lowest *m/z* to highest with a single fragment per row of the table. A fragment is indicated by its *m/z*, relative intensity, structural annotation number, and annotation-specific intensities in parentheses, respectively. When multiple structural annotations are predicted for a single fragment, the relative intensities of each are provided sequentially and tab-separated in parentheses (see *m/z* 150.0105033 in Fig. 2 for an example). Fragment structural annotations are defined using SMILES at the end of each prediction (not pictured in the example Fig. 2). The files “spectra_EI-MS.dat” (“Predicted EI-MS Spectra of CompTox Chemicals Dashboard Structures”, data available at 10.23645/epacomptox.7776212.v1)^23^, “spectra_ESI-MSMS-neg.dat” (“Predicted MS/MS Spectra in ESI-negative mode of CompTox Chemicals Dashboard Structures”, data available at 10.23645/epacomptox.7776212.v1)^23^, and “spectra_ESI-MSMS-pos.dat” (Predicted MS/MS Spectra in ESI-positive mode of CompTox Chemicals Dashboard Structures”, data available at 10.23645/epacomptox.7776212.v1)^23^ contain all predictions consecutively within the.dat files. Entries are separated by the presence of the chemical structure metadata fields described above.

### SQL database

In addition to raw files containing the predicted MS/MS spectra, data was stored in a SQL relational database (“Database of Predicted Spectra of CompTox Chemicals Dashboard Structures”, data available at 10.23645/epacomptox.7776212.v1)^23^. Each chemical structure processed through CFM-ID resulted in MS/MS data from multiple ionization modes and collision energies. This collection of data (chemical structure, identifier, fragments and intensities) is identified as a single job.

**Fig. 3:**
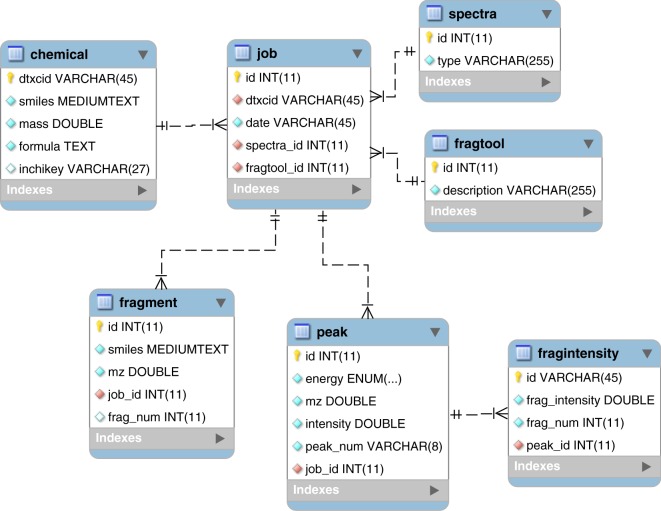
Enhanced Entity Relationship (EER) Diagram of the MySQL database created to host predicted MS/MS data generated using CFM-ID.

These relationships are reflected in the Enhanced Entity Relationship (EER) Diagram (see Fig. 3) and provided as an SQL schema in a separate file (“Database Schema File of Predicted Spectra of CompTox Chemicals Dashboard Structures”, data available at 10.23645/epacomptox.7776212.v1)^23^. The “chemical” table contains the list of all processed chemicals, denoted by a unique DTXCID. The “job” table represents the processing of a chemical for a selected spectrum and provides links into the “peak” and “fragment” tables. In addition, the “peak” table is linked to the “fragintensity” table which contains the fragment intensities and structural annotations for a given peak.

Access to the database is made available through a Python script. In addition to querying the database the script is also capable of ranking the matched chemicals according to their cosine dot product score^25,26^. Relevant information, including the mass of the parent ion, the DTXCID of the parent mass, the masses and intensities of the fragments, and the collision energy, are all provided by the querying script to perform the ranking. The MySQL database is accessed through the PyMySQL module in Python. A query is constructed to combine the fragmentation information from different tables, based on an initial search of the mass of the parent ion or the chemical formula. When the mass is searched, an accuracy level (typically within 10 ppm) is provided. The query will then search for all chemicals with masses within the defined accuracy window, and the predicted fragments for all three collision energies are provided. This information is then loaded into a DataFrame using the Pandas^27^ module in Python, and further calculations, including relative intensities, cosine dot product, and ranking of the matched chemicals are performed.

### Chemical metadata

Chemical metadata linked through the DTXCID are provided for all records for which predicted MS/MS spectra exist. An example of chemical metadata for a subset of structures is provided in Table 1. Metadata are provided in the “CFM-ID_metadata_DTXCID.csv” file for the following categories (“Chemical Metadata from the CompTox Chemicals Dashboard Linked to Predicted Spectra”, data available at 10.23645/epacomptox.7776212.v1)^23^:

- DTXCID: the unique DSSTox chemical identifier for the structure
- DTXSID: the unique DSSTox substance identifier
- Preferred Name
- Chemical Abstracts Service Registry Number (CASRN)
- MS-Ready Molecular Formula
- MS-Ready Monoisotopic Mass
- MS-Ready SMILES
- Data Sources: the number of data sources in which a chemical is found within EPA’s DSSTox database
- PubMed Reference Count: the number of references within PubMed associated with a given DTXSID queried using Medical Subject Heading (MeSH) annotation
- PubChem Data Sources: the number of data sources within PubChem for a given DTXSID
- CPDat Product Occurrence Count: the number of unique consumer products in which a chemical has been reported^24^
- Presence in the following lists: NORMAN Merged Suspect List: SusDat^28,29^, STOFF-IDENT Database (https://www.lfu.bayern.de/stoffident/#!home), ToxCast^30^.

**Table 1:** Chemical metadata for a subset of chemicals defined by DTXCID.

DTXCID	DTXSID	PREFERRED_NAME	CASRN	MS_READY_MOLECULAR_FORMULA	MS_READY_MONOISOTOPIC_MASS	MS_READY_SMILES	DATA_SOURCES	NUMBER_OF_PUBMED_ARTICLES	PUBCHEM_DATA_SOURCES	CPDAT_COUNT	SUSDAT	STOFFIDENT	TOXCAST
DTXCID8068549	DTXSID00146058	Tetrazepam	10379-14-3	C16H17ClN2O	288.10294	CN1C2=C(C=C(Cl)C=C2)C(=NCC1=O)C1=CCCCC1	26	85	30	5	Y	Y	-
DTXCID0077853	DTXSID00155362	N(4)-Acetylsulfadiazine	127-74-2	C12 H12N4O3S	292.06301	CC(=O)NC1=CC=C(C=C1)S(=O)(=O)NC1=NC=CC=N1	19	7	51	—	Y	Y	—
DTXCID20208682	DTXSID00173127	N-L-Alanyl-L-alanine	1948-31-8	C6H12N2O3	160.08479	CC(N)C(=O)NC(C)C(O)=O	13	172	53	—	Y	—	—
DTXCID10104684	DTXSID00182193	(8,8′-Bi-2H-1-benzopyran)-2,2′-dione, 4,4′,7,7′-tetramethoxy-5,5′-dimethyl-, (+)- (9CI)	27909-08-6	C24H22O8	438.13147	COC1=CC(=O)OC2=C1C(C)=CC(OC)=C2C1=C(OC)C=C(C)C2=C1OC(=O)C=C2OC	7	—	15	—	Y	—	—
DTXCID40105487	DTXSID00182996	Methyl naphthoate	28804-90-2	C12H10O2	186.06808	COC(=O)C1=CC=CC2=CC=CC=C12	12	—	49	—	Y	—	—
DTXCID60122353	DTXSID00199862	Dioxypyramidon	519-65-3	C13H17N3O3	263.12699	CN(C)C(=O)C(=O)N(N(C)C(C)=O)C1=CC=CC=C1	12	—	18	—	Y	Y	—
DTXCID6022	DTXSID0020022	Acifluorfen	50594-66-6	C14H7ClF3NO5	360.99648	OC(=O)C1=C(C=CC(OC2=CC=C(C=C2Cl)C(F)(F)F)=C1)[N+]([O-])=O	65	50	74	36	Y	Y	Y
DTXCID5074	DTXSID0020074	Gabapentin	60142-96-3	C9H17NO2	171.12593	NCC1(CC(O)=O)CCCCC1	53	3053	177	29	Y	Y	-
DTXCID9076	DTXSID0020076	Amitrole	61-82-5	C2H4N4	84.043596	NC1=NNC=N1	88	7089	200	28	Y	—	Y
DTXCID00209011	DTXSID0020107	Aspartame	22839-47-0	C14H18N2O5	294.12157	COC(=O)C(CC1=CC=CC=C1)NC(=O)C(N)CC(O)=O	59	862	111	84	Y	Y	Y
DTXCID40232	DTXSID0020232	Caffeine	58-08-2	C8H10N4O2	194.08038	CN1C=NC2=C1C(=O)N(C)C(=O)N2C	116	21207	287	2384	Y	Y	Y
DTXCID80311	DTXSID0020311	Monuron	150-68-5	C9H11ClN2O	198.05599	CN(C)C(=O)NC1=CC=C(Cl)C=C1	72	24	77	47	Y	Y	Y
DTXCID20440	DTXSID0020440	Dichlorprop	120-36-5	C9H8Cl2O3	233.98505	CC(OC1=C(Cl)C=C(Cl)C=C1)C(O)=O	77	89	105	73	Y	Y	Y
DTXCID80442	DTXSID0020442	2,4-Dichlorophenoxyacetic acid	94-75-7	C8H6Cl2O3	219.9694	OC(=O)COC1=C(Cl)C=C(Cl)C=C1	115	2614	175	173	Y	Y	Y
DTXCID00446	DTXSID0020446	Diuron	330-54-1	C9H10Cl2N2O	232.01702	CN(C)C(=O)NC1=CC(Cl)=C(Cl)C=C1	110	1257	132	252	Y	Y	Y

## Technical Validation

The reliability and accuracy of predicted MS/MS spectra using CFM-ID have been reviewed and validated in multiple publications^19,20,26^ and subsequent applications^5,9^. Therefore, to verify the accuracy and ultimate utility of the present work, simple and small scale comparisons were conducted between predictions generated using the CFM-ID web application (http://cfmid.wishartlab.com/) and our own implementation of the command line tools. MS/MS spectra for three randomly selected structures in all three ionization types (for a total of nine comparison points) were predicted using each method and saved as text files (Supplementary Files 1 and 2). Supplementary Files 1 and 2 present the output data copied from each source for a single collision energy for each ionization type. The CFM-ID web application truncates the number of predicted spectra output^19^ and as such slight differences in predicted relative intensities and total number of spectra between the web application and our implementation were expected. As expected, comparison indicated exact output matching for smaller structures with fewer fragments (e.g. DTXCID107640/OC(CC(O)=O)C(O)=O) and highly similar outputs when spectra were truncated in the web application output (e.g. DTXCID00224961/NC(N)=NCCCC(NC=O)C(O)=O). In the instances where exact replication was not observed, only the relative intensities differ and do so by ~1%. Predicted fragments in all cases have identical *m/z* values between the two sources, indicating agreement between our implementation and the web application output.

Chemical metadata validation results from structural curation efforts and mapping within DSSTox between structural identifiers. To certify appropriate mapping between predicted spectra, chemical structures, and selected chemical metadata, a semi-automated process is conducted to link unique chemical identifiers with curated data. Mappings between MS-Ready DTXCIDs and linked DTXSIDs are stored in a structure relationship mapping table to facilitate access to pertinent chemical metadata associated with a DTXSID. The DSSTox database structure, MS-Ready linkages, and chemistry data have been previously described and validated^18^.

## Usage Notes

Predicted MS/MS data are often used by researchers to compare an unidentified chemical (observed via HRMS) to a list of potential candidate chemicals. Empirically collected MS/MS data are scored against predicted spectra of a list of candidate chemicals to identify the best match. Spectral match scores provide an important piece of confirmatory data towards ultimate compound identification. A match score can be calculated between two sets of peaks using a variety of mathematical formulas^25,26,31^, any of which can be executed with simple queries of the present data. The most common use case will require a user to first query the database (or exported file converted to a data frame, for example) based on the parent mass or molecular formula of interest (i.e. observed via HRMS experimentation). The resulting set of structures from the defined search parameters will contain predicted MS/MS data. These data must then be parsed, and ionization mode identified (if desired) in order to match and ultimately score peaks. Here we provide the means to conduct these searches using code developed in Python and match scores computed using the cosine dot product (https://github.com/USEPA/CFM-ID_generation_of_CompTox_Chemicals_Dashboard_Structures_Paper). The matched chemicals, along with their fragments and the corresponding intensities at specific collision energies, are fed into a Python script that matches predicted with experimental spectra. A mass accuracy window (within a few ppm) is needed to search for matches between the fragments of the two spectra. Fragments that fall within this accuracy window are considered a match and are used in the final calculation of the cosine dot product score. The calculation as implemented in our work is computed at all three predicted energy levels. The matched chemicals are then ranked based on individual energy scores or their sum, depending on the user’s preference.

Another potentially less common use case with these data involves a user interested in the predicted MS/MS spectra of a single structure. In this case again, a simple query of the database using structural identifiers will return the desired result. Ultimately, users will be able to conduct the aforementioned queries and calculations within a web interface via the CompTox Chemicals Dashboard. Development is in progress as of December 2018 and the prototype (with the scoring algorithm implemented in Java) enables users to input a mass or formula along with observed MS/MS data and query the database for matches. Users with experience in Python and/or with data requiring customization of the match code will find the Python code of greater value while the Dashboard represents the most accessible means with which to access these data.

Additional chemical metadata linked via structural identifiers presents more options for users to increase the certainty of identifications of unknowns. These data can be accessed directly by querying the full comma-separated export using candidate chemicals. Once retrieved, data source counts associated with candidate chemicals can be used to rank within the set: the greater the number of data sources the more likely the chemical would occur in a sample^6,32^. Preliminary research indicates that data sources contained within DSSTox merged with CFM-ID match scores substantially boosts the number of correct identifications from unknowns. Optimization of combined scoring metrics is under development for implementation via the Dashboard.

## Supplementary Information

### ISA-Tab metadata file

Download metadata file

### Supplementary information

Supplementary File 1

Supplementary File 2

## Data Availability

All code for predicting the MS/MS spectra including model parameters and settings are available via http://sourceforge.net/projects/cfm-id. Additional scripts used to implement the prediction algorithm and query the compiled database are available on GitHub (https://github.com/USEPA/CFM-ID_generation_of_CompTox_Chemicals_Dashboard_Structures_Paper).
